# Increased left ventricular myocardial extracellular volume is associated with longer cardiopulmonary bypass times, biventricular enlargement and reduced exercise tolerance in children after repair of Tetralogy of Fallot

**DOI:** 10.1186/s12968-016-0290-x

**Published:** 2016-10-26

**Authors:** Eugénie Riesenkampff, Wietske Luining, Mike Seed, Paweena Chungsomprasong, Cedric Manlhiot, Bernadette Elders, Brian W. McCrindle, Shi-Joon Yoo, Lars Grosse-Wortmann

**Affiliations:** 1 Department of Pediatrics, Division of Cardiology, Labatt Family Heart Centre, The Hospital for Sick Children, University of Toronto, 555 University Avenue, Toronto, ON M5G 1X8 Canada; 2 Department of Diagnostic Imaging, The Hospital for Sick Children, University of Toronto, 555 University Avenue, Toronto, ON M5G 1X8 Canada

**Keywords:** Tetralogy of Fallot, Diffuse fibrosis, T1 mapping

## Abstract

**Background:**

Unfavorable left ventricular (LV) remodelling may be associated with adverse outcomes after Tetralogy of Fallot (TOF) repair. We sought to assess T1 cardiovascular magnetic resonance (CMR) markers of diffuse LV myocardial fibrosis in children after TOF repair, and associated factors.

**Methods:**

In this prospective, cross-sectional study, native (=non-contrast) T1 times and extracellular volume fraction (ECV) were quantified in the LV myocardium using CMR. Results were related to ventricular volumes and function, degree of pulmonary regurgitation, as well as surgical characteristics, and exercise capacity.

**Results:**

There was no difference in native T1 times or ECV between 31 TOF patients (age at CMR 13.9 ± 2.4 years, 19 male) and 15 controls (age at CMR 13.4 ± 2.6 years, 7 male). Female TOF patients had higher ECVs than males (25.2 ± 2.9 % versus 22.7 ± 3.3 %, *p* < 0.05). In the patient group, higher native T1 and ECV correlated with higher Z-Scores of right and left ventricular end-diastolic volumes, but not with reduced left and right ventricular ejection fraction or higher pulmonary regurgitation fraction. Longer cardiopulmonary bypass and aortic cross clamp times at surgery correlated with increased native T1 times and ECVs (*r* = 0.48, *p* < 0.05 and *r* = 0.65, *p* < 0.01, respectively). Maximum workload (percent of predicted for normal) correlated inversely with ECV (*r* = -0.62, *p* < 0.05). Higher native T1 times correlated with worse LV longitudinal (*r* = 0.50, *p* < 0.05) and mid short axis circumferential strain (*r* = 0.38, *p* < 0.05).

**Conclusions:**

As compared to controls, TOF patients did not express higher markers of diffuse fibrosis. Longer cardiopulmonary bypass and aortic cross clamp times at surgery as well as biventricular enlargement and reduced exercise tolerance are associated with markers of diffuse myocardial fibrosis after TOF repair. Female patients have higher markers of diffuse myocardial fibrosis than males.

## Background

While the vast majority of patients after repair for Tetralogy of Fallot (TOF) survive well into their adult years [1], there is progressive decline not only in right ventricular (RV), but also in left ventricular (LV) function [2]. Even though TOF is primarily a ‘right-sided’ heart disease, LV dysfunction is well recognized and predicts adverse outcomes after TOF repair [3, 4]. Myocardial fibrosis has been associated with LV dysfunction in a number of cardiac conditions, including adult patients with TOF [5, 6]. It is conceivable that myocardial injury triggered by cardiopulmonary bypass and ischemia-reperfusion sets in motion a profibrotic cascade.

Longitudinal relaxation or T1 times and extracellular volume fraction (ECV), measured with cardiovascular magnetic resonance (CMR), have been established as non-invasive markers of diffuse myocardial fibrosis [7]. Both have been validated against histology in the LV of adult patients with different types of heart diseases [8]. However, only very preliminary data exist on the extent and clinical importance of diffuse LV myocardial fibrosis in pediatric TOF patients [6].

The aim of this study was to measure native T1 times and ECV in children after TOF repair, and to understand their potential origin and clinical significance.

## Methods

The cohort in this prospective, single-center study included patients with repaired TOF who underwent a non-sedated clinical contrast-enhanced CMR between March 2014 and January 2015. Patients with 'TOF type' double outlet right ventricle were included. Patients with pulmonary atresia and no major aortopulmonary collateral arteries were also included. Patients with pulmonary valve replacement following the initial complete repair and before CMR were excluded.

Immediately prior to the CMR, blood was drawn to measure the hematocrit. Patients’ records were reviewed for demographic, clinical and surgical details. QRS duration was determined if an ECG was available within 6 months of the CMR. Echocardiograms performed within 6 months from CMR were reviewed for degree of tricuspid valve insufficiency, estimated right ventricular systolic pressure as assessed via the tricuspid regurgitation jet, tricuspid annular plane systolic excursion (TAPSE) and the peak gradient across the right ventricular outflow tract. Exercise test results were reviewed if performed within 6 months from CMR.

Patients who underwent CMR for screening purposes for palpitations, non-specific chest pain, or family history of arrhythmogenic right ventricular cardiomyopathy served as controls, as long as the CMR and all other tests were normal.

### CMR

#### Image acquisition

All CMR examinations were performed on a 1.5 Tesla scanner (Magnetom Avanto, Siemens AG Healthcare Sector, Erlangen, Germany) using a phased-array multi-channel surface receiver coil. A modified Look-Locker inversion recovery sequence (MOLLI) [9] was used to measure T1 times of myocardium and blood. The sequence consisted of two inversion-recovery prepared ECG synchronized Look-Locker experiments with inversion pulses at 120 ms and 200 ms after the R-spike, respectively, as well as 5 and 3 single-shot images after these inversion pulses. The pause (measured in beats) between the two Look-Locker experiments was adjusted according to the patients’ heart rate: three heart beats up to a heart rate of 80 bpm, four heart beats for heart rates > 80 and < 100 bpm, and five heart beats for patients with a heart rate of 100 bpm and higher. Other sequence parameters were as follows: Repetition and echo times 2.7 ms and 1.1 ms respectively; in-plane resolution 2.0x1.3 mm, slice-thickness 8 mm; flip angle 35°. Images were acquired in diastole in mid-ventricular and basal short axis (SA) planes before and 15 min after the intravenous administration of 0.1 mmol/kg gadobenate dimeglumine (Multihance®, Bracco Imaging, Montreal, Quebec, Canada). Breathholds were used in cooperative patients; all other patients were scanned during free breathing. A cine 4-chamber slice and a stack of multiphase SA slices were acquired in the steady state free precession technique to measure LV and RV volumes, as described previously [10]. Blood flow volumes were measured in the main, right and left pulmonary arteries using phase contrast flow velocity mapping in the usual clinical fashion [11].

The presence of LV late gadolinium enhancement (LGE) was determined qualitatively on standard long-axis (4-chamber, 2-chamber and 3-chamber) and SA images using phase-sensitive inversion-recovery acquisitions ≥ 10 min after administration of the contrast agent (same injection as described above).

#### Image processing

Using the 8 raw images of the MOLLI sequence, native and post-contrast T1 times were measured by an experienced observer (ER), using commercially available software (CVI42, Circle Cardiovascular Imaging, Calgary, AB, Canada). Regions of interest were drawn in mid and basal SA in the interventricular septum, the LV free wall, the entire LV myocardium and in the LV blood pool, avoiding papillary muscles. If necessary, contours were adjusted in each phase. Myocardial contours comprised the central 2/3 of the myocardium, in order to avoid possible signal averaging with signal from blood or pericardial fat. For assessment of intra- and interobserver variability, all contours were redrawn by the primary as well as by a second observer in 15 randomly selected TOF patients.

ECV was calculated using the following formula [12]:

$$ \mathrm{E}\mathrm{C}\mathrm{V}=\left(1-\mathrm{hematocrit}\right) \times \frac{\left(\frac{1}{\mathrm{T}1\ \mathrm{myocardium}\ \mathrm{p}\mathrm{ost}} - \frac{1}{\ \mathrm{T}1\ \mathrm{myocardium}\ \mathrm{p}\mathrm{r}\mathrm{e}}\right)}{\left(\frac{1}{\mathrm{T}1\ \mathrm{blood}\ \mathrm{p}\mathrm{ost}} - \frac{1}{\mathrm{T}1\ \mathrm{blood}\ \mathrm{p}\mathrm{r}\mathrm{e}}\right)} $$

RV and LV volumes were measured from the stack of SA cine images in systole and diastole with calculation of ejection fraction. LV muscle mass was measured in diastole (Medis Q-Mass 7.6, Leiden, The Netherlands). RV muscle mass was measured in the patient group in diastole. Results were indexed to body surface area, and Z-scores for ventricular volumes and ejection fractions were calculated using published normative data [13].

Cardiomechanics were measured using steady state free precession cine imaging and CMR feature tracking with commercially available software (Image Arena Version 4.6, TomTec Imaging Systems, Unterschleissheim, Germany). The following parameters were assessed: Peak LV longitudinal strain and strain rate from 4-chamber view; circumferential strain and strain rate at the LV mid and basal SA levels, as well as LV torsion, calculated from rotation and ventricular dimensions as described elsewhere [14]. Pulmonary artery flow was quantified using Q-Flow 5.6 (Medis, Leiden, The Netherlands).

### Statistical analysis

Data is described as means with standard deviations or medians with interquartile ranges (IQR) or frequencies as appropriate. Comparisons between groups (TOF patients and controls) and TOF subgroups were performed using Fisher’s exact test for categorical variables, and Student’s t-test for continuous variables, assuming unequal variance between samples (Satterthwaite methods). Associations of age, gender, heart rate, BSA, hematocrit and QRS-duration with T1 mapping results in TOF patients and controls were modelled in linear regression models (maximum likelihood method for parameter estimation). Pearson correlations were computed to assess associations between continuous variables. Variables with associations with fibrosis markers were entered into multivariable regression models for native T1 times and ECV. Intra- and interobserver variability for native T1 times and ECV were assessed using the Bland-Altman method. All statistical analyses were performed using SAS Version 9.3 (SAS statistical software, Cary NC). *P* values < 0.05 were considered significant.

## Results

Thirty-six patients after TOF repair met the inclusion criteria. Five patients were excluded from the analysis: Two patients had undergone late repair at the age of 8 years and were regarded as non-representative of the current surgical era; one patient had a VSD patch leak with a residual shunt of Qp:Qs 1:5. T1 mapping images were non-diagnostic in two patients.

Characteristics of the remaining 31 patients in comparison to 15 controls and surgical information of the patients are summarized in Table 1.

**Table 1:** Characteristics of patient and controls and surgical information of patients

	Patients (*n* = 31)	Controls (*n* = 15)	*p*
Age at CMR [years]	13.9 ± 2.4	13.4 ± 2.6	0.54
Gender [male; n (%)]	19 (61 %)	7 (44 %)	0.53
BSA [m^2^]	1.47 ± 0.27	1.55 ± 0.30	0.41
Heart rate [bpm]	73 ± 11	76 ± 11	0.31
Hematocrit	0.44 ± 0.04	0.42 ± 0.04	0.16
QRS-duration [ms]^a^	123 ± 24	87 ± 8	<0.001
Surgical information			
Patient age at repair [months]	6.3 (3.0–10.7)	n.a.	n.a.
Time since repair at CMR [years]	13.2 ± 2.2	n.a.	n.a.
TOF type [n (%)]		n.a.	n.a.
TOF TOF with pulmonary atresia	25 (81 %) 6 (19 %)		
Type of repair [n (%)]^b^		n.a.	n.a.
Transannular patch Valve sparing procedure or valved conduit	13 (43 %) 17 (57 %)		
Bypass time [minutes]^c^	136 ± 48	n.a.	n.a.
Cross clamp time [minutes]^c^	67 ± 19	n.a.	n.a.

Data are given as mean ± standard deviation, median (and interquartile range), or frequencies, as appropriate

*Abbreviations*: *BSA* body surface area, *CMR* cardiovascular magnetic resonance, *n.a*. not applicable, *TOF* Tetralogy of Fallot

^a^QRS-duration from ECG was available in 26 patients and 11 controls

^b^Type of repair unknown in one patient. One patient had a palliation prior to corrective surgery

^c^Cardiopulmonary bypass and aortic cross clamp times from 23 patients

### Ventricular volumes and function

Results of CMR and echocardiography are shown in Table 2. As expected, the RVs of patients were enlarged as compared to controls, and biventricular ejection fraction was reduced. Male TOF patients had larger ventricular volumes as compared to females (indexed right and left ventricular enddiastolic and endsystolic volumes: LVEDVi 95 ± 12 versus 85 ± 9 ml/m^2^; LVESVi 44 ± 8 versus 37 ± 7 ml/m^2^; RVEDVi 159 ± 44 versus 129 ± 23 ml/m^2^; RVESVi 82 ± 26 ml/m^2^ versus 64 ± 14 ml/m^2^, all *p* < 0.05) and a higher RV muscle mass (44 ± 9 versus 32 ± 3 g/m^2^, *p* < 0.001). There were no gender differences in ejection fraction of both ventricles and LV muscle mass in the TOF group. Female patients had been slightly younger at the time of repair (median age 3.8 (IQR 0.7–6.7) versus 7.4 (IQR 5.4–12.6 months, *p* < 0.05).

**Table 2:** Results from CMR and echocardiography

	Patients (*n* = 31)	Controls (*n* = 15)	*p*
CMR Ventricular volumetry and pulmonary flow			
RV enddiastolic volume [ml/m^2^]	148 ± 40	93 ± 13	<0.001
RV endsystolic volume [ml/m^2^]	75 ± 23	43 ± 8	<0.001
RV muscle mass [g/m^2^]	39.4 ± 9.4	n.a.	n.a.
RV ejection fraction [%]	50 ± 5	53 ± 3	<0.05
LV enddiastolic volume [ml/m^2^]	91 ± 12	89 ± 11	0.50
LV endsystolic volume [ml/m^2^]	41 ± 8	36 ± 6	<0.05
LV muscle mass [g/m^2^]	46 ± 7	49 ± 9	0.29
LV ejection fraction [%]	55 ± 6	59 ± 4	<0.01
Pulmonary regurgitation [%]	33 ± 16	n.a.	n.a.
Z-Scores			
RV enddiastolic volume	3.42 (1.52–5.29)	−0.11 (−1.17–0.68)	<0.001
RV endsystolic volume	3.65 (1.52–4.51)	−0.05 (−0.33–0.98)	<0.001
RV ejection fraction	−1.52 (−2.23– −0.69)	−1.04 (−1.29– −0.39)	<0.05
LV enddiastolic volume	0.62 ( −0.10–1.36)	0.41 (−0.55–1.59)	0.60
LV endsystolic volume	1.57 (−0.75–2.30)	1.08 (−0.31–1.65)	<0.05
LV muscle mass	−1.89 (−2.90– −0.94)	−0.96 (−2.43–0.36)	0.24
LV ejection fraction	−1.29 (−2.51– −0.80)	−0.32 (−1.41–0.41)	<0.01
Echocardiography			
RVSP [mmHg], *n* = 19	44 ± 15	n.a.	n.a.
RVOT-gradient [mmHg], *n* = 29	32 ± 18	n.a.	n.a.
TAPSE [mm], *n* = 19	16.5 ± 3.5	n.a.	n.a.
Grade of tricuspid insufficiency [n (%)]		n.a.	n.a.
Trivial Mild	17 (55 %) 14 (45 %)		

Data are given as mean ± standard deviation, median (and interquartile range), or frequencies, as appropriate

*Abbreviations*: *LV* left ventricle, *PT* pulmonary trunk, *RV* right ventricle, *RVOT* right ventricular outflow tract, *RVSP* right ventricular systolic pressure, *TAPSE* tricuspid annular plane systolic excursion

In TOF patients RVEDVi correlated with LVEDVi (*r* = 0.52, *p* < 0.005). There was no correlation between RV and LV ejection fractions or between their respective Z-scores.

Mid SA circumferential strain was weaker in the patient group as compared to controls (−21.5 ± 3.8 % versus −24.1 ± 3.3 %, *p* < 0.05). Other cardiomechanical values (basal and mid circumferential strain rate and basal mid circumferential strain) did not show significant differences.

Echocardiographic data was available in 29 patients within 6 months of CMR (Table 2). The median time difference between echocardiography and CMR was 20 days (IQR 0-50 days).

### Cardiopulmonary exercise test and QRS duration

QRS durations in TOF patients and controls are provided in Table 1. Exercise test results were available in 15 patients within 6 months from CMR. The median time difference of both investigations was 39 days (IQR 19–60 days). Expressed as percent of predicted for normal, maximal workload was 96 ± 12 % and oxygen uptake at anaerobic threshold was 93 ± 30 %. Males (*n* = 9) had a higher percentage of predicted oxygen uptake at anaerobic threshold than females (110 ± 23 % versus 69 ± 20 %, *p* < 0.05).

### Native T1 and ECV

Native T1 times and ECV are listed in Table 3. There were no significant differences between patients and controls for all regions analysed within the LV. In TOF patients, septal values were higher as compared to the free wall in mid and basal SA (native T1 times basal SA 989 ± 43 ms versus 963 ± 52 ms, *p* < 0.05; ECV mid SA 24.3 ± 3.7 % versus 22.3 ± 3.8 %, *p* < 0.05). These differences were not present in the control group.

**Table 3:** ECV and native T1 results

		Patients	*n*	Controls	*n*	*p*
Native T1 (ms)						
Entire left ventricle	mid	974 ± 37	29	986 ± 29	15	0.26
	base	982 ± 37	31	998 ± 25	15	0.10
Interventricular septum	mid	990 ± 49	31	1000 ± 39	15	0.44
	base	989 ± 43	31	999 ± 28	15	0.34
Left ventricular free wall	mid	970 ± 50	31	989 ± 46	15	0.20
	base	963 ± 52	30	983 ± 30	15	0.11
Extracellular volume (%)						
Entire left ventricle	mid	23.6 ± 3.3	28	23.4 ± 3.0	14	0.84
	base	24.0 ± 3.2	28	22.9 ± 2.6	13	0.25
Interventricular septum	mid	24.3 ± 3.7	30	22.8 ± 2.7	14	0.13
	base	23.7 ± 3.3	29	23.1 ± 2.8	13	0.57
Left ventricular free wall	mid	22.3 ± 3.8	30	23.1 ± 2.7	14	0.43
	base	22.3 ± 3.9	27	21.5 ± 2.4	13	0.45

Data are given as mean ± standard deviation. The varying numbers *n* of available values for the different measurements is attributed to image quality, i.e., image quality of some native or post contrast T1 maps was reduced in parts

Female TOF patients had higher values as compared to male TOF patients for native T1 in the basal interventricular septum (1009 ± 41 versus 976 ± 40 ms, *p* < 0.05) and for ECV in all parts analysed (e.g., mid SA entire LV 25.2 ± 2.9 % versus 22.7 ± 3.3 %, *p* < 0.05). No patient had LV LGE.

### Intra- and interobserver variability

**Fig. 1:**
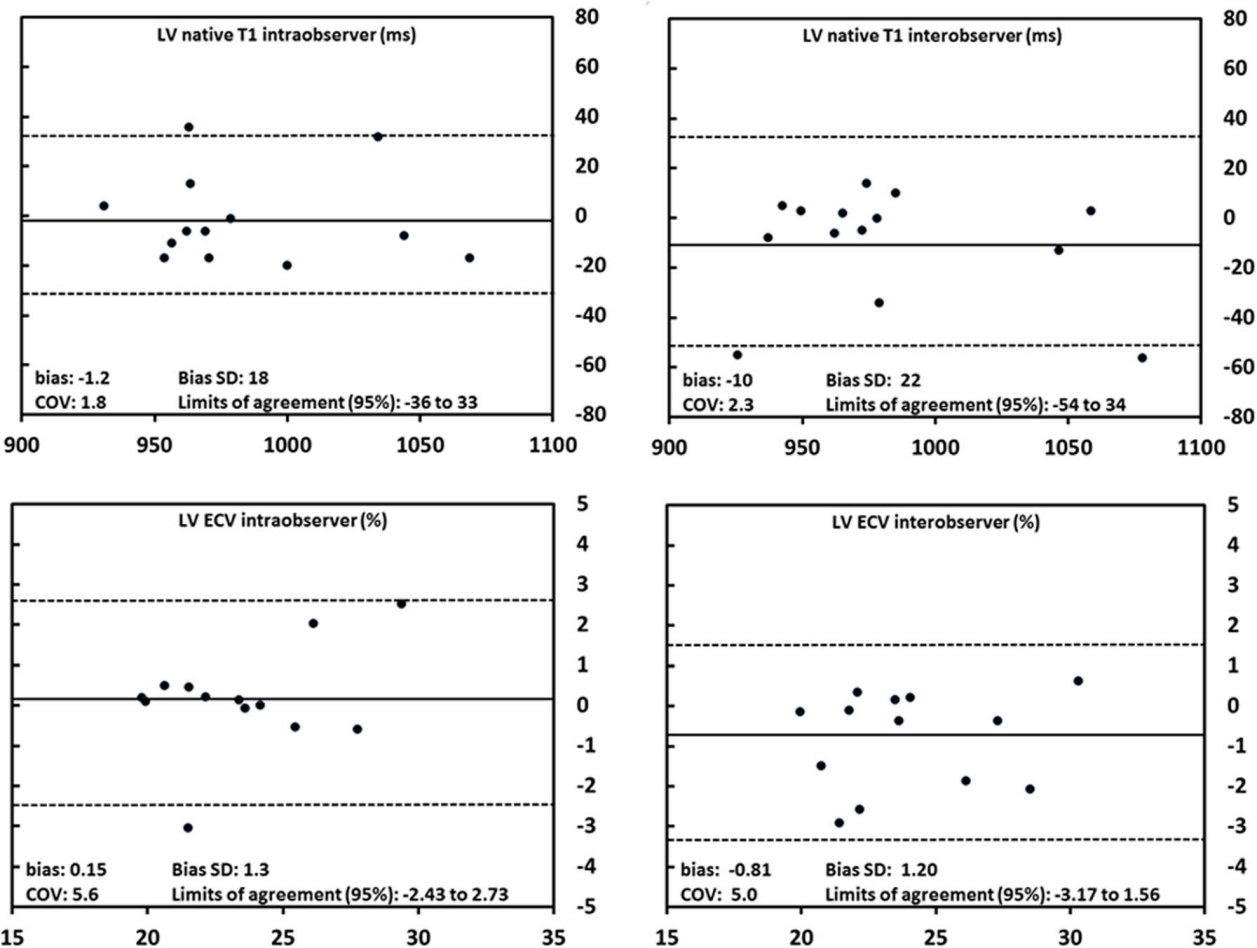
Inter- and intraobserver variabilities of native T1 and ECV. Inter- and intraobserver variabilities of native T1 and ECV measured in the entire LV in mid short axis. Abbreviations: COV = coefficient of variation; ECV = extracellular volume; LV = left ventricle; SD = standard deviation

Mean bias, coefficient of variation, standard deviation and limits of agreement of inter- and intraobserver variability for native T1 times and ECV in mid SA for the entire LV are shown in Fig. 1.

### Associations between T1 and ECV and demographic and surgical factors

Age at surgery correlated inversely with native T1 and ECV in the IVS (*r* = −0.504, *p* < 0.005 and *r* = −0.447, *p* < 0.05, respectively) and entire LV (*r* = −0.420, *p* < 0.02 and *r* = −0.468, *p* < 0.01, respectively) at the base. Longer cardiopulmonary bypass time correlated with elevated native T1 time of the IVS (*r* = 0.48, *p* < 0.05), and with higher ECV of the entire LV (*r* = 0.46, *p* < 0.05) and IVS (*r* = 0.48, *p* < 0.05), all at the mid SA level. Longer aortic cross clamp time correlated with higher native T1 of the LV free wall in mid SA (*r* = 0.65, *p* < 0.01). In a multivariable analysis of age at surgery, cardiopulmonary bypass and aortic cross clamp times, longer bypass and cross clamp times were independently associated with elevated native T1 and ECV (in the regions described above). Patients who had undergone either a valve sparing or valved conduit repair had lower fibrosis markers than patients who had a transannular patch repair (native T1 entire LV mid SA 960 ± 28 versus 991 ± 40 ms, *p* < 0.05; ECV entire LV basal SA 22.6 ± 2.7 % versus 26.0 ± 3.0 %, *p* < 0.01). Expectedly, pulmonary regurgitant volume was higher in the transannular patch group (2.53 ± 1.30 versus 1.48 ± 1.14 L/min/m^2^, *p* < 0.05).

Maximal workload (percent of predicted for normal) correlated inversely with ECV of the entire LV in basal SA (*r* = −0.62, *p* < 0.05) and ECV of the basal LV free wall (*r* = −0.68, *p* < 0.05).

### Associations between T1 and ECV and ventricular function and exercise tolerance

Indexed ventricular volumes did not correlate with T1 mapping results. However, higher Z-scores of LVEDV, RVEDV and RVESV correlated with higher ECV in mid SA in all regions analysed (e.g., for entire LV *r* = 0.39, *p* < 0.05, *r* = 0.46, *p* < 0.01, and *r* = 0.43, *p* < 0.05, respectively). There were no correlations of T1 mapping results with LV or RV ejection fraction or pulmonary regurgitation. Neither T1 times nor ECV correlated with RV systolic pressure or TAPSE by echocardiography. Worse (i.e., less negative) left ventricular 4-chamber longitudinal strain correlated with higher native T1 times (entire LV mid SA *r* = 0.50, *p* < 0.05; IVS mid SA *r* = 0.55, *p* < 0.05; IVS basal SA *r* = 0.50, *p* < 0.05). At the midventricular SA level, there was a weak correlation of higher native T1 times of the LV free wall and worse circumferential strain (*r* = 0.38, *p* < 0.05). There were no further correlations between T1 mapping (T1 and ECV) and myocardial strain, strain rate or torsion.

## Discussion

Adult survivors after TOF repair have evidence of increased LV ECV, quantified by CMR T1 mapping [5, 15, 16]. In an earlier pilot study, we found preliminary evidence for a higher LV fibrosis burden in pediatric TOF patients [6]. To the best of our knowledge, the current study is the first prospective investigation on the degree, potential etiology, and clinical significance of CMR markers of myocardial fibrosis in young patients after TOF repair. The principal novel findings of this study are:

I. On average, children and adolescents do not express CMR markers of increased amounts of LV myocardial fibrosis.
II. Higher ECV is associated with larger LV and RV volumes.
III. Longer cardiopulmonary bypass and aortic cross clamp times and younger age at primary surgical repair are associated with higher native T1 times and ECV.
IV. The transannular patch approach is associated with higher markers of fibrosis than valve sparing techniques and valved RV to pulmonary artery conduits.
V. Higher markers of diffuse fibrosis are associated with myocardial dysfunction and reduced cardiopulmonary exercise tolerance.

Although primarily a ‘right heart’ condition, LV dysfunction is not uncommon in TOF and a risk factor for adverse outcomes [3, 4]. The interactions between RV and LV physiology are well recognized, but the pathophysiology of them is incompletely understood: Unfavourable RV hemodynamics and septal physiology have been proposed as mediators of RV/LV ‘cross-talk’ [17, 18]. In the present study LVEDV correlated with RVEDV. The association of LV ECV not only with LV but also with RV volume make it tempting to speculate that ventricular interaction also exists on a tissue level. In fact, Friedberg et al. recently showed experimentally that abnormal RV loading leads not only to right, but also to left ventricular fibrosis, probably via the transforming growth factor pathway [19]. Native T1 times and ECV were higher in the IVS than in the LV free wall in patients, but not in controls. Although speculative, the higher fibrosis markers in the IVS could mean that ventriculoventricular interactions affect mainly the part of the myocardium that is directly adjacent to the RV with which it shares fibres. Studies that include reliable ECV measurements in the RV will be necessary to understand regional variations of fibrotic remodeling.

Our previous pilot study suggested increased LV fibrosis in children with repaired TOF [6]. However, those conclusions rested on a small number of patients who had post-contrast T1 mapping only, an approach which is now considered inferior to ECV and native T1 as markers of fibrosis [7]. In the current larger prospective study there were no differences of native T1 times and ECV between patients and controls. This is in agreement with studies in adults with ischemic and valvar heart disease which consistently report significant overlap of health and disease [20]. In contrast to our study Broberg et al. found higher ECVs in adult TOF patients than in controls [5, 15]. Importantly, their patients were, on average, 20 years older than ours. As a consequence, Broberg’s patients had had more time for adverse LV remodelling and adverse clinical outcome to occur; even so, 37 out of their 52 TOF patients [15] had ECVs in the normal range and the TOF subgroup had the lowest average ECV of all congenital heart disease patients [5]. The larger RVs and LVs in Broberg’s adult TOF patients as compared to our pediatric patients are further hallmarks of remodeling which, in our study, are associated with CMR markers of fibrosis. In addition, the patients in Broberg’s study may represent a different ‘surgical era’: Our results support the influence of surgical strategy on late myocardial remodelling: Firstly, patients after transannular patch repair, the strategy which prevailed during the surgical era in Broberg’s study, had higher ECV in our study than patients who received a valved RV outflow tract. The reasons for this difference remain speculative. Whether it is the RV volume loading which is more severe in patients after transannular patch or whether the strategy of transannular patch is a surrogate for more severe preoperative outflow tract obstruction is unclear [21]. Secondly, surgical procedural factors like longer cardiopulmonary bypass and cross clamp times were related to increased markers of myocardial fibrosis. An association of these parameters with fibrotic myocardial remodelling more than a decade after repair of congenital heart disease has not previously been appreciated, although we know from endomyocardial biopsies in patients after heart transplantation that longer graft ischemia time is associated with more ventricular scarring [22]. The results from this study underscore the importance of optimal cardioprotection and of minimizing the duration of cardiopulmonary bypass and aortic cross clamp times. The pathophysiolgy of fibrotic remodelling in congenital heart disease, including TOF, is largely unknown. Besides myocardial injury from surgery genetic upregulation of collagen synthesis, chronic hypoxia, and hemodynamic disturbances have all been named as possible promoters of fibrosis [21].

Recently, a study in adults with repaired TOF found ECV to be associated with arrhythmia [16]. An exploration of the pediatric TOF cohort with CMRs at our institution revealed no deaths and a very low incidence of ventricular tachyarrhythmia (unpublished data) which means that a risk-prediction analysis with T1 relaxometry in young patients has to rely on surrogate parameters of adverse clinical status and outcomes, such as exercise tolerance. Indeed, ECV correlated with lower maximum workload. Girls after TOF repair expressed higher markers of fibrosis than boys, which has been shown in adult TOF patients [15, 16]. It is interesting to note that girls also had larger ventricles, lower ejection fractions and weaker exercise tolerance, corroborating previous reports [23, 24]. Together, these data suggest a more rapid deterioration of cardiovascular health in female TOF patients.

Whether myocardial fibrosis causes impaired myocardial contractility remains to be clarified. In the patients studied here CMR markers of fibrosis were not related to LV ejection fraction, but higher ECV and native T1 were associated with decreased LV myocardial contractility, as evidenced by lower longitudinal and circumferential strain. These associations suggest that fibrotic remodelling does, indeed, translate into loss of myocardial function, even though the health of LVs of juvenile TOF patients is relatively preserved.

### Limitations

T1 and ECV in different regions of the heart correlated with the various clinical parameters, likely due to the limited sample size, and the region with the highest diagnostic yield remains to be identified. Likewise, it is unclear whether ECV offers a diagnostic advantage over non-contrast native T1. It would be preferred to be able to avoid the use of gadolinium in light of concerns regarding possible gadolinium deposition in the brain [25] and nephrogenic systemic fibrosis [26]. Shortening the inversion times within the MOLLI sequence is advisable for post-contrast T1 times, taking into account the shorter T1 recovery kinetics [27]. Although a motion-correction algorithm was employed and the eight MOLLI raw images were reviewed for misregistration, this approach remains inferior to acquisitions during breathholding [28]. Whether or not ECV can be measured reliably in the RV is under debate [5]. Plymen and colleagues reported that even in systemic RVs of adult patients a reliable assessment of ECV was not possible [29]. Chen et al., in their study of adult TOF patients, were able to measure RV ECV with acceptable reproducibility [16]. In our experience the quantification of post-contrast T1 in the RV of children without myocardial hypertrophy often fails. We, therefore, did not include RV ECV in our analysis.

## Conclusions

On average, children and adolescents after TOF repair do not express increased native T1 or ECV. Nonetheless, elevated CMR markers of LV fibrosis are associated with biventricular enlargement and LV myocardial dysfunction. Longer cardiopulmonary bypass and aortic cross clamp times appear to plant the seed for chronic fibrotic LV remodeling. Longitudinal studies are needed to inform us about the contribution of CMR T1 mapping to the prediction of outcomes in TOF patients.

## Abbreviations

CMR: Cardiovascular magnetic resonance
ECV: Extracellular volume
IQR: Interquartile ranges
LGE: Late gadolinium enhancement
LV: Left ventricle
LVEDV(i): (indexed) Left ventricular end-diastolic volume
LVESV(i): (indexed) Left ventricular end-systolic volume
MOLLI: Modified Look-Locker inversion recovery
RV: Right ventricle
RVEDV(i): (indexed) Right ventricular end-diastolic volume
RVESV(i): (indexed) Right ventricular end-systolic volume
SA: Short axis
TAPSE: Tricuspid annular plane systolic excursion
TOF: Tetralogy of Fallot
